# Bis{μ-*N*-[(*E*)-4-benz­yloxy-2-oxidobenzyl­idene]-4-nitro­benzene­carbo­hydrazidato}bis­[di­aqua­nickel(II)] di­methyl­formamide tetra­solvate

**DOI:** 10.1107/S1600536814010150

**Published:** 2014-05-17

**Authors:** Bibitha Joseph, M. Sithambaresan, M. R. Prathapachandra Kurup, Seik Weng Ng

**Affiliations:** a Department of Applied Chemistry, Cochin University of Science and Technology, Kochi 682 022, India; b Department of Chemistry, Faculty of Science, Eastern University, Sri Lanka, Chenkalady, Sri Lanka; c Department of Chemistry, University of Malaya, 50603 Kuala Lumpur, Malaysia; d Chemistry Department, King Abdulaziz University, PO Box 80203 Jeddah, Saudi Arabia

## Abstract

The molecule of the title complex, [Ni_2_(C_21_H_15_N_3_O_5_)_2_(H_2_O)_4_]·4C_3_H_7_NO, is located on an inversion centre. This results in a dimeric Ni^II^ complex, with the two Ni^II^ atoms bridged by phenolate O atoms. The tridentate ligand is chelated to each Ni^II^ atom *via* one N and two O atoms of the imino­late form of the hydrazide moiety, which has the same conformation as the free ligand. The coordination geometry around each Ni^II^ ion is slightly distorted octa­hedral. A supra­molecular three-dimensional architecture is created by dominant inter­molecular O—H⋯N, O—H⋯O and C—H⋯O hydrogen-bonding inter­actions. These are augmented by two C—H⋯π inter­actions and a π–π inter­action with a centroid–centroid distance of 3.681 (2) Å.

## Related literature   

For biological applications of hydrazinecarboxamide and its derivatives, see: Lakshmi *et al.* (2011 ▶); Prasanna & Kumar (2013 ▶); Singh *et al.* (2007 ▶); Naseema *et al.* (2010 ▶). For the synthesis of related compounds, see: Joseph *et al.* (2013 ▶). For related structures, see: Joseph *et al.* (2012 ▶); Raj & Kurup (2007 ▶). For standard bond lengths, see: Allen *et al.* (1987 ▶).
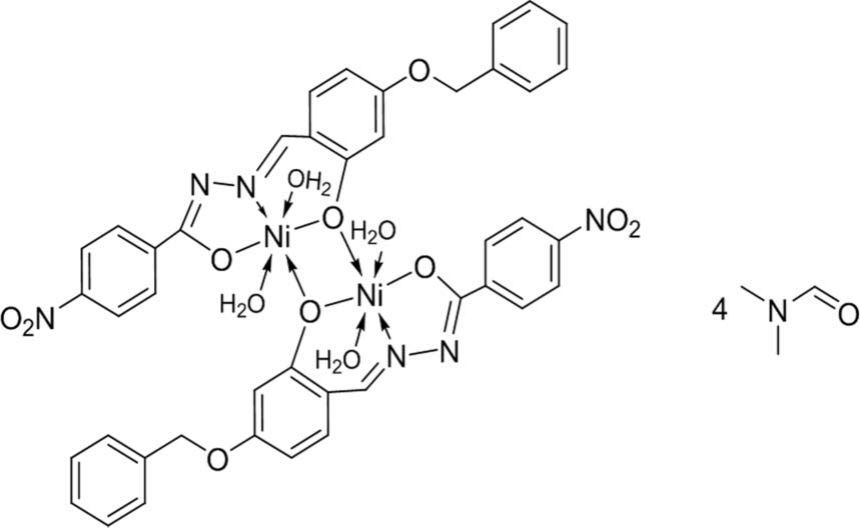


## Experimental   

### 

#### Crystal data  

[Ni_2_(C_21_H_15_N_3_O_5_)_2_(H_2_O)_4_]·4C_3_H_7_NO
*M* *_r_* = 1260.55
Triclinic, 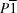

*a* = 8.4939 (3) Å
*b* = 12.5451 (6) Å
*c* = 14.6717 (6) Å
α = 81.662 (2)°
β = 75.613 (1)°
γ = 79.442 (1)°
*V* = 1480.56 (11) Å^3^
*Z* = 1
Mo *K*α radiation
μ = 0.72 mm^−1^
*T* = 293 K
0.40 × 0.25 × 0.20 mm

#### Data collection  

Bruker Kappa APEXII CCD diffractometer
Absorption correction: multi-scan (*SADABS*; Bruker, 2004 ▶) *T*_min_ = 0.763, *T*_max_ = 0.870
11081 measured reflections
6571 independent reflections 6785
4713 reflections with *I* > 2σ(*I*)
*R*_int_ = 0.025

#### Refinement  

*R*[*F* ^2^ > 2σ(*F* ^2^)] = 0.048
*wR*(*F* ^2^) = 0.138
*S* = 1.02
6785 reflections
383 parameters
H-atom parameters constrained
Δρ_max_ = 0.44 e Å^−3^
Δρ_min_ = −0.42 e Å^−3^

### 

Data collection: *APEX2* (Bruker, 2004 ▶); cell refinement: *APEX2* and *SAINT* (Bruker, 2004 ▶); data reduction: *SAINT* and *XPREP* (Bruker, 2004 ▶); program(s) used to solve structure: *SHELXS97* (Sheldrick, 2008 ▶); program(s) used to refine structure: *SHELXL97* (Sheldrick, 2008 ▶); molecular graphics: *ORTEP-3 for Windows* (Farrugia, 2012 ▶) and *DIAMOND* (Brandenburg, 2010 ▶); software used to prepare material for publication: *SHELXL97* and *publCIF* (Westrip, 2010 ▶).

## Supplementary Material

Crystal structure: contains datablock(s) I, global. DOI: 10.1107/S1600536814010150/fj2672sup1.cif

Structure factors: contains datablock(s) I. DOI: 10.1107/S1600536814010150/fj2672Isup2.hkl

CCDC reference: 1001219

Additional supporting information:  crystallographic information; 3D view; checkCIF report

## Figures and Tables

**Table 1:** Hydrogen-bond geometry (Å, °) *Cg*4 and *Cg*5 are the centroids of the C1–C6 and C9–C14 rings, respectively.

*D*—H⋯*A*	*D*—H	H⋯*A*	*D*⋯*A*	*D*—H⋯*A*
O1*W*—H11⋯O6	0.84	2.00	2.702 (4)	140
O1*W*—H12⋯O7	0.84	1.98	2.674 (4)	139
O2*W*—H21⋯O1*W* ^i^	0.84	2.40	2.862 (2)	115
O2*W*—H22⋯N2^ii^	0.84	2.43	2.908 (3)	117
C2—H2⋯O3^i^	0.93	2.36	3.217 (3)	153
C18—H18⋯O7^iii^	0.93	2.43	3.257 (6)	147
C26—H26*B*⋯O5^iv^	0.96	2.56	3.320 (8)	136
C22—H22*A*⋯*Cg*4	0.93	2.95	3.440 (5)	115
C23—H23*B*⋯*Cg*5^v^	0.96	2.94	3.89 (8)	172

Symmetry codes: (i) 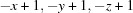; (ii) 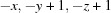; (iii) 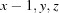; (iv) 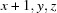; (v) 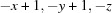.

**Fig. 1.**
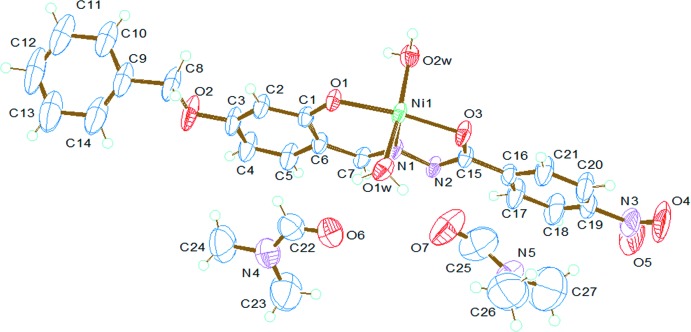
ORTEP view of the unique part of the compound, drawn with 50% probability displacement ellipsoids for the non-H atoms.

**Fig. 2.**
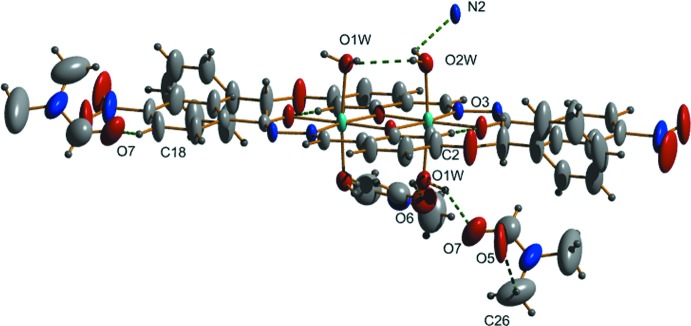
Graphical representation showing hydrogen bonding interactions in the crystal structure of [C42H38N6Ni2O14]·4(C3H7NO).

**Fig. 3.**
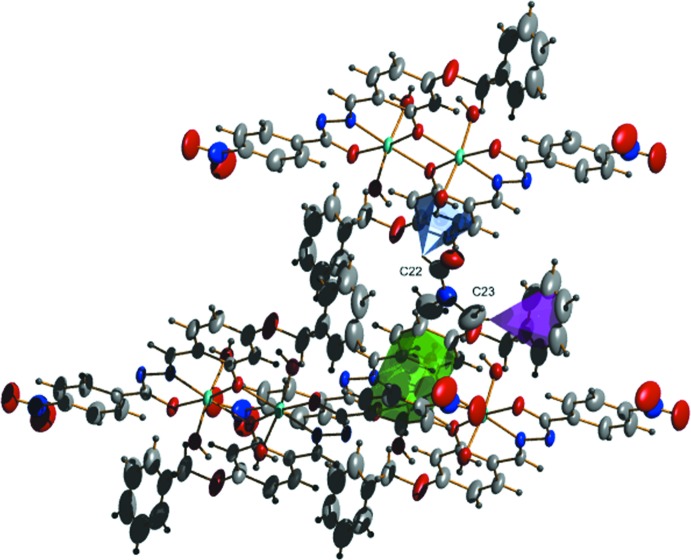
C–H···π and π···π interactions found in the title compound.

**Fig. 4.**
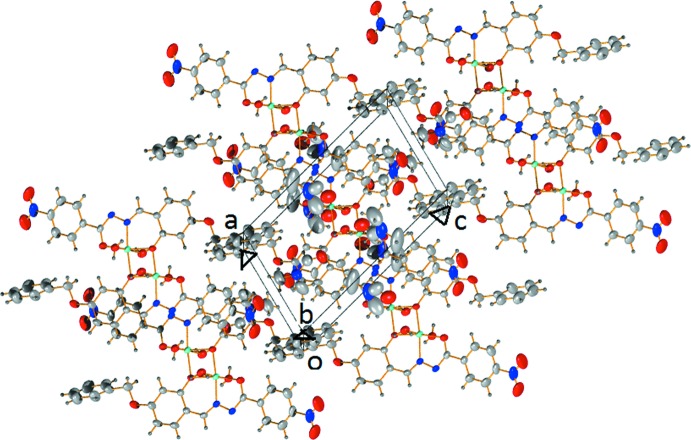
A view of the unit cell along b axis.

## supplementary crystallographic information

### 1. Comment

Hydrazones and their nickel complexes have been shown to possess a diverse range of biological activities (Lakshmi *et al.*, 2011; Prasanna & Kumar, 2013). In most cases, the metal complexes show more antibacterial activity compared to their parent ligands (Singh *et al.*, 2007). They also have applications in chemical processes like non-linear optics, sensors *etc* (Naseema *et al.*, 2010).

The title complex [C_42_H_38_N_6_Ni_2_O_14_]·4(C_3_H_7_NO) has a dimeric structure. This is generated by the unique part of the Ni^II^ complex (Fig. 1) bridging two Ni atoms through phenolate O atoms. The molecule adopts an *E* configuration with respect to C7═N1 bond and the tridentate ligand has its coordinating entities disposed in a *cis* fashion to each other. The Ni atom in the complex is N,O,O' chelated by the iminolate form of the hydrazide ligand. The C7═N1 [1.272 (3) Å] and C15–O3 [1.282 (3) Å] bond distances are very close to the formal C═N and C–O bond lengths (Allen *et al.*, 1987) respectively confirming the azomethine bond formation and the coordination *via* iminolate form. The coordination geometry around each Ni^II^ ion is octahedral with a slight distortion. The hydrazide moiety of the free ligand is coordinated to each Ni atom *via* iminolate form of the hydrazide moiety without changing its configuration (Joseph *et al.*, 2012; Raj & Kurup, 2007; Joseph *et al.*, 2013).

There are seven O–H···N, O–H···O and C–H···O intermolecular (classical and non-classical) hydrogen bonding interactions (Table 1), which interconnect the neighbouring complex and the solvent DMF molecules with D···A distances of 2.702 (4), 2.674 (4), 2.862 (3), 2.908 (3), 3.217 (3), 3.257 (6) and 3.320 (8) Å (Fig. 2). Two C–H···π interactions with H···*Cg* distances of 3.440 (5) and 3.894 (7) Å progressing along *c* axis and a π···π interaction (Fig. 3) with a *Cg*···*Cg* distance of 3.681 (2) Å progressing along *b* axis also support the dominant intermolecular hydrogen bonding interactions to establish a supramolecular three-dimensional network in the crystal system. Fig. 4 shows the packing diagram of the title compound along *b* axis.

### 2. Experimental

The title complex was prepared by adapting a reported procedure (Joseph *et al.*, 2013) by mixing hot methanolic solutions of *N*'-[(*E*)-4-benzyloxy-2-hydroxybenzylidene]-4-nitrobenzohydrazide dimethylformamide monosolvate (0.464 g, 1 mmol) and Ni(OAc)_2_·4H_2_O (0.248 g, 1 mmol) for 4 h. On cooling, brown colored product formed were collected, washed with few drops of methanol and dried over P_4_O_10_*in vacuo*. Single crystals of the title compound suitable for X-ray analysis were obtained by recrystallization from a mixture of methanol and dimethylformamide (1:1 *v*/*v*). The compound was obtained in 65% yield (0.857 g).

### 3. Refinement

All H atoms on C were placed in calculated positions, guided by difference maps, with C—H bond distances of 0.93–0.97 Å. H atoms were assigned *U*_iso_(H) values of 1.2Ueq(carrier). Omitted owing to bad disagreement was reflections (0 - 1 1), (0 1 1), (0 0 1) and (0 1 0).

### Crystal data

**Table:**

[Ni_2_(C_21_H_15_N_3_O_5_)_2_(H_2_O)_4_]·4C_3_H_7_NO	*Z* = 1
*M**_r_* = 1260.55	*F*(000) = 660
Triclinic, *P*1	*D*_x_ = 1.414 Mg m^−^^3^
Hall symbol: -P 1	Mo *K*α radiation, λ = 0.71073 Å
*a* = 8.4939 (3) Å	Cell parameters from 3205 reflections
*b* = 12.5451 (6) Å	θ = 2.9–26.1°
*c* = 14.6717 (6) Å	µ = 0.72 mm^−^^1^
α = 81.662 (2)°	*T* = 293 K
β = 75.613 (1)°	Block, brown
γ = 79.442 (1)°	0.40 × 0.25 × 0.20 mm
*V* = 1480.56 (11) Å^3^	

### Data collection

**Table:**

Bruker Kappa APEXII CCD diffractometer	6571 independent reflections
Radiation source: fine-focus sealed tube	4713 reflections with *I* > 2σ(*I*)
Graphite monochromator	*R*_int_ = 0.025
Detector resolution: 8.33 pixels mm^-1^	θ_max_ = 27.5°, θ_min_ = 2.6°
ω and φ scan	*h* = −11→6
Absorption correction: multi-scan (*SADABS*; Bruker, 2004)	*k* = −16→16
*T*_min_ = 0.763, *T*_max_ = 0.870	*l* = −19→16
11081 measured reflections	

### Refinement

**Table:**

Refinement on *F*^2^	Primary atom site location: structure-invariant direct methods
Least-squares matrix: full	Secondary atom site location: difference Fourier map
*R*[*F*^2^ > 2σ(*F*^2^)] = 0.048	Hydrogen site location: inferred from neighbouring sites
*wR*(*F*^2^) = 0.138	H-atom parameters constrained
*S* = 1.02	*w* = 1/[σ^2^(*F*_o_^2^) + (0.0721*P*)^2^ + 0.2776*P*] where *P* = (*F*_o_^2^ + 2*F*_c_^2^)/3
6785 reflections	(Δ/σ)_max_ = 0.001
383 parameters	Δρ_max_ = 0.44 e Å^−^^3^
0 restraints	Δρ_min_ = −0.42 e Å^−^^3^

### Special details

**Table:**

Geometry. All e.s.d.'s (except the e.s.d. in the dihedral angle between two l.s. planes) are estimated using the full covariance matrix. The cell e.s.d.'s are taken into account individually in the estimation of e.s.d.'s in distances, angles and torsion angles; correlations between e.s.d.'s in cell parameters are only used when they are defined by crystal symmetry. An approximate (isotropic) treatment of cell e.s.d.'s is used for estimating e.s.d.'s involving l.s. planes.
Refinement. Refinement of *F*^2^ against ALL reflections. The weighted *R*-factor *wR* and goodness of fit *S* are based on *F*^2^, conventional *R*-factors *R* are based on *F*, with *F* set to zero for negative *F*^2^. The threshold expression of *F*^2^ > σ(*F*^2^) is used only for calculating *R*-factors(gt) *etc*. and is not relevant to the choice of reflections for refinement. *R*-factors based on *F*^2^ are statistically about twice as large as those based on *F*, and *R*- factors based on ALL data will be even larger.

### Fractional atomic coordinates and isotropic or equivalent isotropic displacement parameters (Å^2^)

**Table:**

	*x*	*y*	*z*	*U*_iso_*/*U*_eq_	
Ni1	0.32132 (3)	0.56165 (3)	0.52457 (2)	0.03177 (13)	
O1	0.47991 (19)	0.48838 (15)	0.41614 (12)	0.0316 (4)	
O2	0.6760 (3)	0.3770 (3)	0.10470 (16)	0.0811 (10)	
O3	0.1376 (2)	0.64291 (17)	0.61866 (14)	0.0427 (5)	
O4	−0.5294 (5)	0.9447 (4)	0.8836 (3)	0.1297 (16)	
O5	−0.6793 (4)	0.9197 (3)	0.7950 (3)	0.1240 (15)	
O6	0.3857 (4)	0.7887 (3)	0.2882 (2)	0.0846 (9)	
O7	0.3202 (5)	0.8970 (3)	0.5412 (3)	0.1077 (12)	
O1W	0.4206 (2)	0.70844 (16)	0.46434 (15)	0.0427 (5)	
H11	0.4496	0.7089	0.4052	0.064*	
H12	0.3486	0.7627	0.4783	0.064*	
O2W	0.2508 (2)	0.40630 (17)	0.58160 (14)	0.0418 (5)	
H21	0.3131	0.3740	0.6167	0.063*	
H22	0.2585	0.3688	0.5372	0.063*	
N1	0.1453 (2)	0.59582 (18)	0.45394 (15)	0.0317 (5)	
N2	−0.0044 (2)	0.65070 (19)	0.50159 (16)	0.0342 (5)	
N3	−0.5476 (4)	0.9086 (3)	0.8164 (3)	0.0864 (12)	
N4	0.4309 (4)	0.7733 (3)	0.1324 (2)	0.0737 (9)	
N5	0.1458 (6)	1.0356 (3)	0.6066 (3)	0.1009 (15)	
C1	0.4525 (3)	0.4821 (2)	0.33231 (18)	0.0318 (6)	
C2	0.5816 (3)	0.4342 (3)	0.2632 (2)	0.0423 (7)	
H2	0.6845	0.4099	0.2766	0.051*	
C3	0.5579 (4)	0.4229 (3)	0.1763 (2)	0.0557 (10)	
C4	0.4067 (4)	0.4603 (3)	0.1528 (2)	0.0636 (11)	
H4	0.3913	0.4531	0.0935	0.076*	
C5	0.2821 (4)	0.5078 (3)	0.2195 (2)	0.0536 (9)	
H5	0.1808	0.5328	0.2043	0.064*	
C6	0.2981 (3)	0.5210 (2)	0.30952 (19)	0.0353 (6)	
C7	0.1548 (3)	0.5754 (2)	0.37016 (19)	0.0360 (6)	
H7	0.0612	0.5976	0.3460	0.043*	
C8	0.8333 (4)	0.3326 (4)	0.1248 (3)	0.0764 (14)	
H8A	0.8215	0.2755	0.1772	0.092*	
H8B	0.8817	0.3890	0.1418	0.092*	
C9	0.9403 (4)	0.2870 (4)	0.0367 (3)	0.0756 (13)	
C10	1.0033 (5)	0.1770 (4)	0.0401 (3)	0.0836 (14)	
H10	0.9775	0.1317	0.0960	0.100*	
C11	1.1050 (5)	0.1345 (5)	−0.0403 (4)	0.1038 (19)	
H11A	1.1476	0.0607	−0.0388	0.125*	
C12	1.1420 (5)	0.2034 (6)	−0.1226 (4)	0.117 (2)	
H12A	1.2106	0.1747	−0.1762	0.141*	
C13	1.0813 (6)	0.3122 (6)	−0.1277 (3)	0.119 (2)	
H13	1.1064	0.3569	−0.1840	0.143*	
C14	0.9792 (5)	0.3550 (5)	−0.0451 (3)	0.107 (2)	
H14	0.9385	0.4291	−0.0463	0.128*	
C15	0.0093 (3)	0.6707 (2)	0.5843 (2)	0.0341 (6)	
C16	−0.1380 (3)	0.7329 (3)	0.6438 (2)	0.0400 (7)	
C17	−0.2891 (4)	0.7543 (3)	0.6213 (3)	0.0636 (11)	
H17	−0.3014	0.7294	0.5672	0.076*	
C18	−0.4227 (4)	0.8118 (4)	0.6775 (3)	0.0750 (13)	
H18	−0.5244	0.8258	0.6615	0.090*	
C19	−0.4044 (4)	0.8476 (3)	0.7558 (2)	0.0592 (10)	
C20	−0.2586 (5)	0.8284 (4)	0.7814 (3)	0.0735 (12)	
H20	−0.2484	0.8538	0.8358	0.088*	
C21	−0.1248 (4)	0.7701 (3)	0.7250 (2)	0.0640 (11)	
H21A	−0.0242	0.7558	0.7422	0.077*	
C22	0.4572 (5)	0.7448 (4)	0.2167 (3)	0.0716 (11)	
H22A	0.5382	0.6853	0.2235	0.086*	
C23	0.3069 (8)	0.8619 (6)	0.1161 (4)	0.140 (3)	
H23A	0.2534	0.8919	0.1745	0.209*	
H23B	0.2276	0.8364	0.0914	0.209*	
H23C	0.3560	0.9170	0.0714	0.209*	
C24	0.5236 (7)	0.7171 (5)	0.0517 (3)	0.1112 (18)	
H24A	0.5928	0.6536	0.0726	0.167*	
H24B	0.5903	0.7649	0.0082	0.167*	
H24C	0.4490	0.6955	0.0208	0.167*	
C25	0.1844 (8)	0.9403 (5)	0.5711 (5)	0.117 (2)	
H25	0.0983	0.9047	0.5696	0.140*	
C26	0.2699 (10)	1.0969 (5)	0.6094 (5)	0.154 (3)	
H26A	0.3686	1.0741	0.5638	0.231*	
H26B	0.2919	1.0848	0.6715	0.231*	
H26C	0.2327	1.1731	0.5948	0.231*	
C27	−0.0180 (10)	1.0771 (7)	0.6473 (7)	0.231 (6)	
H27A	−0.0907	1.0479	0.6201	0.347*	
H27B	−0.0347	1.1551	0.6353	0.347*	
H27C	−0.0405	1.0565	0.7143	0.347*	

### Atomic displacement parameters (Å^2^)

**Table:**

	*U*^11^	*U*^22^	*U*^33^	*U*^12^	*U*^13^	*U*^23^
Ni1	0.01855 (17)	0.0461 (2)	0.0311 (2)	0.00660 (13)	−0.00672 (12)	−0.01827 (16)
O1	0.0220 (8)	0.0457 (11)	0.0290 (9)	0.0055 (7)	−0.0087 (7)	−0.0184 (8)
O2	0.0481 (13)	0.146 (3)	0.0468 (14)	0.0433 (15)	−0.0216 (11)	−0.0589 (16)
O3	0.0269 (9)	0.0638 (14)	0.0375 (11)	0.0146 (9)	−0.0110 (8)	−0.0266 (10)
O4	0.112 (3)	0.172 (4)	0.078 (2)	0.064 (3)	0.000 (2)	−0.070 (3)
O5	0.0563 (18)	0.172 (4)	0.112 (3)	0.052 (2)	0.0070 (18)	−0.047 (3)
O6	0.102 (2)	0.088 (2)	0.0554 (17)	−0.0048 (17)	−0.0103 (16)	−0.0082 (16)
O7	0.107 (3)	0.082 (2)	0.141 (3)	0.0190 (19)	−0.040 (2)	−0.057 (2)
O1W	0.0339 (9)	0.0425 (12)	0.0517 (13)	0.0051 (8)	−0.0118 (9)	−0.0151 (10)
O2W	0.0310 (9)	0.0549 (13)	0.0406 (11)	−0.0009 (9)	−0.0097 (8)	−0.0127 (10)
N1	0.0199 (9)	0.0416 (13)	0.0329 (12)	0.0058 (9)	−0.0055 (8)	−0.0154 (10)
N2	0.0183 (9)	0.0465 (14)	0.0357 (13)	0.0085 (9)	−0.0059 (9)	−0.0154 (11)
N3	0.068 (2)	0.098 (3)	0.063 (2)	0.040 (2)	0.0104 (18)	−0.018 (2)
N4	0.077 (2)	0.086 (3)	0.059 (2)	−0.0139 (19)	−0.0130 (17)	−0.0139 (19)
N5	0.122 (3)	0.056 (2)	0.092 (3)	0.019 (2)	0.013 (3)	−0.009 (2)
C1	0.0273 (12)	0.0396 (16)	0.0297 (14)	0.0007 (11)	−0.0080 (10)	−0.0129 (12)
C2	0.0291 (13)	0.062 (2)	0.0350 (15)	0.0114 (13)	−0.0103 (11)	−0.0213 (14)
C3	0.0397 (16)	0.090 (3)	0.0365 (17)	0.0191 (16)	−0.0108 (13)	−0.0355 (18)
C4	0.0478 (17)	0.105 (3)	0.0419 (18)	0.0241 (18)	−0.0237 (14)	−0.041 (2)
C5	0.0361 (15)	0.085 (3)	0.0425 (18)	0.0159 (15)	−0.0205 (13)	−0.0292 (18)
C6	0.0293 (12)	0.0473 (17)	0.0312 (14)	0.0022 (11)	−0.0098 (11)	−0.0154 (13)
C7	0.0231 (12)	0.0492 (18)	0.0375 (15)	0.0063 (11)	−0.0126 (11)	−0.0153 (13)
C8	0.0428 (18)	0.128 (4)	0.055 (2)	0.037 (2)	−0.0175 (16)	−0.050 (2)
C9	0.0441 (18)	0.130 (4)	0.052 (2)	0.030 (2)	−0.0181 (16)	−0.050 (2)
C10	0.052 (2)	0.113 (4)	0.084 (3)	0.016 (2)	−0.007 (2)	−0.054 (3)
C11	0.062 (3)	0.133 (5)	0.117 (4)	0.017 (3)	−0.006 (3)	−0.081 (4)
C12	0.055 (3)	0.209 (7)	0.091 (4)	0.021 (3)	−0.006 (3)	−0.098 (5)
C13	0.076 (3)	0.200 (7)	0.067 (3)	0.041 (4)	−0.012 (2)	−0.052 (4)
C14	0.075 (3)	0.167 (5)	0.064 (3)	0.054 (3)	−0.020 (2)	−0.049 (3)
C15	0.0235 (12)	0.0398 (16)	0.0362 (15)	0.0016 (11)	−0.0006 (11)	−0.0135 (12)
C16	0.0293 (13)	0.0493 (18)	0.0359 (16)	0.0061 (12)	−0.0020 (11)	−0.0111 (14)
C17	0.0347 (15)	0.094 (3)	0.060 (2)	0.0211 (17)	−0.0116 (15)	−0.040 (2)
C18	0.0344 (16)	0.109 (3)	0.073 (3)	0.0261 (19)	−0.0099 (16)	−0.034 (2)
C19	0.0442 (17)	0.068 (2)	0.047 (2)	0.0218 (16)	0.0072 (15)	−0.0148 (18)
C20	0.067 (2)	0.096 (3)	0.049 (2)	0.025 (2)	−0.0089 (18)	−0.036 (2)
C21	0.0402 (17)	0.093 (3)	0.054 (2)	0.0248 (17)	−0.0123 (15)	−0.032 (2)
C22	0.073 (3)	0.071 (3)	0.069 (3)	−0.016 (2)	−0.012 (2)	−0.001 (2)
C23	0.138 (5)	0.181 (7)	0.083 (4)	0.036 (5)	−0.040 (4)	−0.012 (4)
C24	0.130 (4)	0.131 (5)	0.070 (3)	−0.017 (4)	−0.006 (3)	−0.035 (3)
C25	0.102 (4)	0.094 (4)	0.150 (6)	0.002 (3)	−0.032 (4)	−0.015 (4)
C26	0.242 (9)	0.095 (5)	0.125 (6)	−0.042 (5)	−0.029 (6)	−0.015 (4)
C27	0.173 (8)	0.199 (9)	0.211 (10)	0.069 (7)	0.072 (7)	0.010 (7)

### Geometric parameters (Å, º)

**Table:**

Ni1—N1	1.974 (2)	C8—C9	1.505 (4)
Ni1—O1	2.0237 (16)	C8—H8A	0.9700
Ni1—O3	2.0335 (17)	C8—H8B	0.9700
Ni1—O1^i^	2.0456 (16)	C9—C14	1.374 (7)
Ni1—O2W	2.136 (2)	C9—C10	1.383 (6)
Ni1—O1W	2.147 (2)	C10—C11	1.389 (6)
O1—C1	1.323 (3)	C10—H10	0.9300
O1—Ni1^i^	2.0456 (16)	C11—C12	1.383 (8)
O2—C3	1.372 (3)	C11—H11A	0.9300
O2—C8	1.434 (4)	C12—C13	1.366 (8)
O3—C15	1.282 (3)	C12—H12A	0.9300
O4—N3	1.197 (5)	C13—C14	1.416 (6)
O5—N3	1.215 (5)	C13—H13	0.9300
O6—C22	1.225 (5)	C14—H14	0.9300
O7—C25	1.186 (6)	C15—C16	1.494 (3)
O1W—H11	0.8400	C16—C17	1.373 (4)
O1W—H12	0.8400	C16—C21	1.377 (5)
O2W—H21	0.8400	C17—C18	1.377 (4)
O2W—H22	0.8400	C17—H17	0.9300
N1—C7	1.272 (3)	C18—C19	1.345 (5)
N1—N2	1.399 (3)	C18—H18	0.9300
N2—C15	1.311 (3)	C19—C20	1.352 (5)
N3—C19	1.474 (4)	C20—C21	1.384 (4)
N4—C22	1.299 (5)	C20—H20	0.9300
N4—C23	1.421 (6)	C21—H21A	0.9300
N4—C24	1.447 (5)	C22—H22A	0.9300
N5—C25	1.326 (7)	C23—H23A	0.9600
N5—C27	1.405 (7)	C23—H23B	0.9600
N5—C26	1.426 (8)	C23—H23C	0.9600
C1—C2	1.405 (3)	C24—H24A	0.9600
C1—C6	1.417 (3)	C24—H24B	0.9600
C2—C3	1.369 (4)	C24—H24C	0.9600
C2—H2	0.9300	C25—H25	0.9300
C3—C4	1.393 (4)	C26—H26A	0.9600
C4—C5	1.363 (4)	C26—H26B	0.9600
C4—H4	0.9300	C26—H26C	0.9600
C5—C6	1.396 (4)	C27—H27A	0.9600
C5—H5	0.9300	C27—H27B	0.9600
C6—C7	1.440 (3)	C27—H27C	0.9600
C7—H7	0.9300		
			
N1—Ni1—O1	91.70 (8)	C10—C9—C8	119.3 (4)
N1—Ni1—O3	79.10 (8)	C9—C10—C11	119.8 (5)
O1—Ni1—O3	170.70 (7)	C9—C10—H10	120.1
N1—Ni1—O1^i^	171.98 (7)	C11—C10—H10	120.1
O1—Ni1—O1^i^	80.54 (7)	C12—C11—C10	119.1 (5)
O3—Ni1—O1^i^	108.71 (7)	C12—C11—H11A	120.4
N1—Ni1—O2W	91.81 (9)	C10—C11—H11A	120.4
O1—Ni1—O2W	88.30 (7)	C13—C12—C11	122.2 (4)
O3—Ni1—O2W	93.27 (8)	C13—C12—H12A	118.9
O1^i^—Ni1—O2W	85.99 (7)	C11—C12—H12A	118.9
N1—Ni1—O1W	92.89 (9)	C12—C13—C14	118.3 (6)
O1—Ni1—O1W	87.03 (8)	C12—C13—H13	120.9
O3—Ni1—O1W	92.07 (8)	C14—C13—H13	120.9
O1^i^—Ni1—O1W	88.74 (7)	C9—C14—C13	120.0 (5)
O2W—Ni1—O1W	173.47 (7)	C9—C14—H14	120.0
C1—O1—Ni1	126.65 (14)	C13—C14—H14	120.0
C1—O1—Ni1^i^	133.85 (14)	O3—C15—N2	126.2 (2)
Ni1—O1—Ni1^i^	99.46 (7)	O3—C15—C16	116.9 (2)
C3—O2—C8	117.0 (2)	N2—C15—C16	116.9 (2)
C15—O3—Ni1	109.58 (16)	C17—C16—C21	117.7 (3)
Ni1—O1W—H11	109.5	C17—C16—C15	122.6 (3)
Ni1—O1W—H12	109.5	C21—C16—C15	119.7 (2)
H11—O1W—H12	109.5	C16—C17—C18	121.3 (3)
Ni1—O2W—H21	109.5	C16—C17—H17	119.4
Ni1—O2W—H22	109.5	C18—C17—H17	119.4
H21—O2W—H22	109.5	C19—C18—C17	119.2 (3)
C7—N1—N2	117.2 (2)	C19—C18—H18	120.4
C7—N1—Ni1	127.20 (16)	C17—C18—H18	120.4
N2—N1—Ni1	115.54 (16)	C18—C19—C20	122.0 (3)
C15—N2—N1	109.52 (19)	C18—C19—N3	119.2 (3)
O4—N3—O5	123.2 (3)	C20—C19—N3	118.8 (4)
O4—N3—C19	119.1 (4)	C19—C20—C21	118.6 (4)
O5—N3—C19	117.7 (4)	C19—C20—H20	120.7
C22—N4—C23	120.6 (4)	C21—C20—H20	120.7
C22—N4—C24	122.3 (4)	C16—C21—C20	121.2 (3)
C23—N4—C24	117.1 (4)	C16—C21—H21A	119.4
C25—N5—C27	121.3 (7)	C20—C21—H21A	119.4
C25—N5—C26	121.2 (5)	O6—C22—N4	126.3 (4)
C27—N5—C26	117.5 (6)	O6—C22—H22A	116.8
O1—C1—C2	118.7 (2)	N4—C22—H22A	116.8
O1—C1—C6	122.7 (2)	N4—C23—H23A	109.5
C2—C1—C6	118.6 (2)	N4—C23—H23B	109.5
C3—C2—C1	120.8 (2)	H23A—C23—H23B	109.5
C3—C2—H2	119.6	N4—C23—H23C	109.5
C1—C2—H2	119.6	H23A—C23—H23C	109.5
C2—C3—O2	125.0 (3)	H23B—C23—H23C	109.5
C2—C3—C4	121.4 (3)	N4—C24—H24A	109.5
O2—C3—C4	113.7 (3)	N4—C24—H24B	109.5
C5—C4—C3	117.8 (3)	H24A—C24—H24B	109.5
C5—C4—H4	121.1	N4—C24—H24C	109.5
C3—C4—H4	121.1	H24A—C24—H24C	109.5
C4—C5—C6	123.6 (3)	H24B—C24—H24C	109.5
C4—C5—H5	118.2	O7—C25—N5	125.0 (6)
C6—C5—H5	118.2	O7—C25—H25	117.5
C5—C6—C1	117.8 (2)	N5—C25—H25	117.5
C5—C6—C7	116.1 (2)	N5—C26—H26A	109.5
C1—C6—C7	126.0 (2)	N5—C26—H26B	109.5
N1—C7—C6	125.6 (2)	H26A—C26—H26B	109.5
N1—C7—H7	117.2	N5—C26—H26C	109.5
C6—C7—H7	117.2	H26A—C26—H26C	109.5
O2—C8—C9	107.1 (3)	H26B—C26—H26C	109.5
O2—C8—H8A	110.3	N5—C27—H27A	109.5
C9—C8—H8A	110.3	N5—C27—H27B	109.5
O2—C8—H8B	110.3	H27A—C27—H27B	109.5
C9—C8—H8B	110.3	N5—C27—H27C	109.5
H8A—C8—H8B	108.5	H27A—C27—H27C	109.5
C14—C9—C10	120.6 (4)	H27B—C27—H27C	109.5
C14—C9—C8	120.0 (4)		
			
N1—Ni1—O1—C1	3.8 (2)	N2—N1—C7—C6	179.3 (3)
O1^i^—Ni1—O1—C1	−178.2 (3)	Ni1—N1—C7—C6	0.9 (4)
O2W—Ni1—O1—C1	95.6 (2)	C5—C6—C7—N1	−179.4 (3)
O1W—Ni1—O1—C1	−89.0 (2)	C1—C6—C7—N1	−0.7 (5)
N1—Ni1—O1—Ni1^i^	−177.97 (9)	C3—O2—C8—C9	179.7 (4)
O1^i^—Ni1—O1—Ni1^i^	0.0	O2—C8—C9—C14	62.8 (5)
O2W—Ni1—O1—Ni1^i^	−86.21 (8)	O2—C8—C9—C10	−119.4 (4)
O1W—Ni1—O1—Ni1^i^	89.22 (8)	C14—C9—C10—C11	−0.9 (7)
N1—Ni1—O3—C15	0.94 (19)	C8—C9—C10—C11	−178.6 (4)
O1^i^—Ni1—O3—C15	−177.14 (18)	C9—C10—C11—C12	0.2 (7)
O2W—Ni1—O3—C15	−90.27 (19)	C10—C11—C12—C13	−0.3 (8)
O1W—Ni1—O3—C15	93.49 (19)	C11—C12—C13—C14	1.0 (8)
O1—Ni1—N1—C7	−2.0 (3)	C10—C9—C14—C13	1.6 (7)
O3—Ni1—N1—C7	176.7 (3)	C8—C9—C14—C13	179.3 (4)
O2W—Ni1—N1—C7	−90.3 (3)	C12—C13—C14—C9	−1.6 (8)
O1W—Ni1—N1—C7	85.2 (3)	Ni1—O3—C15—N2	0.0 (4)
O1—Ni1—N1—N2	179.60 (17)	Ni1—O3—C15—C16	−179.2 (2)
O3—Ni1—N1—N2	−1.74 (17)	N1—N2—C15—O3	−1.5 (4)
O2W—Ni1—N1—N2	91.25 (18)	N1—N2—C15—C16	177.8 (2)
O1W—Ni1—N1—N2	−93.29 (18)	O3—C15—C16—C17	−170.8 (3)
C7—N1—N2—C15	−176.4 (3)	N2—C15—C16—C17	9.9 (5)
Ni1—N1—N2—C15	2.2 (3)	O3—C15—C16—C21	8.6 (5)
Ni1—O1—C1—C2	175.7 (2)	N2—C15—C16—C21	−170.7 (3)
Ni1^i^—O1—C1—C2	−1.8 (4)	C21—C16—C17—C18	0.6 (6)
Ni1—O1—C1—C6	−4.7 (4)	C15—C16—C17—C18	−180.0 (3)
Ni1^i^—O1—C1—C6	177.80 (19)	C16—C17—C18—C19	0.0 (7)
O1—C1—C2—C3	178.1 (3)	C17—C18—C19—C20	−0.2 (7)
C6—C1—C2—C3	−1.6 (5)	C17—C18—C19—N3	−179.6 (4)
C1—C2—C3—O2	−179.7 (3)	O4—N3—C19—C18	−176.6 (5)
C1—C2—C3—C4	1.3 (6)	O5—N3—C19—C18	3.4 (6)
C8—O2—C3—C2	3.4 (6)	O4—N3—C19—C20	4.0 (7)
C8—O2—C3—C4	−177.6 (4)	O5—N3—C19—C20	−176.0 (4)
C2—C3—C4—C5	−0.6 (6)	C18—C19—C20—C21	0.0 (7)
O2—C3—C4—C5	−179.6 (4)	N3—C19—C20—C21	179.3 (4)
C3—C4—C5—C6	0.1 (6)	C17—C16—C21—C20	−0.9 (6)
C4—C5—C6—C1	−0.4 (5)	C15—C16—C21—C20	179.7 (3)
C4—C5—C6—C7	178.5 (4)	C19—C20—C21—C16	0.6 (7)
O1—C1—C6—C5	−178.6 (3)	C23—N4—C22—O6	−1.4 (8)
C2—C1—C6—C5	1.1 (4)	C24—N4—C22—O6	179.2 (4)
O1—C1—C6—C7	2.7 (5)	C27—N5—C25—O7	−175.0 (7)
C2—C1—C6—C7	−177.6 (3)	C26—N5—C25—O7	1.6 (10)

Symmetry code: (i) −*x*+1, −*y*+1, −*z*+1.

### Hydrogen-bond geometry (Å, º)

Cg4 and Cg5 are the centroids of the C1–C6 and C9–C14 rings, respectively.

**Table:**

*D*—H···*A*	*D*—H	H···*A*	*D*···*A*	*D*—H···*A*
O1*W*—H11···O6	0.84	2.00	2.702 (4)	140
O1*W*—H12···O7	0.84	1.98	2.674 (4)	139
O2*W*—H21···O1*W*^i^	0.84	2.40	2.862 (2)	115
O2*W*—H22···N2^ii^	0.84	2.43	2.908 (3)	117
C2—H2···O3^i^	0.93	2.36	3.217 (3)	153
C18—H18···O7^iii^	0.93	2.43	3.257 (6)	147
C26—H26*B*···O5^iv^	0.96	2.56	3.320 (8)	136
C22—H22*A*···*Cg*4	0.93	2.95	3.440 (5)	115
C23—H23*B*···*Cg*5^v^	0.96	2.94	3.89 (8)	172

Symmetry codes: (i) −*x*+1, −*y*+1, −*z*+1; (ii) −*x*, −*y*+1, −*z*+1; (iii) *x*−1, *y*, *z*; (iv) *x*+1, *y*, *z*; (v) −*x*+1, −*y*+1, −*z*.
